# Balance Disorder Trends in US Adults 2008‐2016: Epidemiology and Functional Impact

**DOI:** 10.1002/oto2.58

**Published:** 2023-06-06

**Authors:** Margaret B. Mitchell, Neil Bhattacharyya

**Affiliations:** ^1^ Department of Otolaryngology‐Head & Neck Surgery Massachusetts Eye & Ear Boston Massachusetts USA

**Keywords:** anxiety, balance disorder, depression, dizziness, epidemiology, vertigo

## Abstract

**Objective:**

To quantify the changes in prevalence and impact of dizziness and balance disorders in adults from 2008 to 2016.

**Study Design:**

Epidemiological survey analysis.

**Setting:**

United States.

**Methods:**

The balance modules of the adult 2008 and 2016 National Health Interview Surveys were examined, and persons reporting dizziness or balance problems identified. The prevalence of balance problems was determined and compared over time, adjusting for age and sex. Among those with balance problems, associated symptoms and self‐reported functional limitations were quantified and compared over time.

**Results:**

In 2016, 36.8 ± 1.0 million (15.5% ± 0.3%) adults reported a balance problem in the past year, versus 24.2 ± 0.7 million (11.% ± 0.3%) in 2008 (*p* < .001). After adjustment for age and sex, this percentage increase remained significant (odds ratio 1.435 [1.332‐1.546], *p* < .001). Among those with balance problems, significantly more patients reported specific issues with feeling: off‐balance (69.4% vs. 65.4%; *p* = .005), faint (48.5% vs. 40.3%; *p* < .001), or vertiginous (45.9% vs. 39.3%; *p* < .001) in 2016 than 2008. More adults experienced anxiety (29.4% vs. 19.4%; *p* < .001) and depression (16.3% vs. 12.9%; *p* = .002) with their balance problems in 2016 than in 2008. In 2016, adults with balance problems were limited in ability to drive motor vehicles (13.0%), exercise (14.4%), or walk downstairs (12.8%). These rates were not significantly different from 2008 (all *p* > .05).

**Conclusion:**

In this nationally representative analysis, we found a significantly increasing prevalence of balance problems and associated psychiatric symptom burden. This merits attention with respect to present and future health care resource allocation.

Dizziness and balance issues are common complaints with a substantial disease burden in the United States; dizziness alone is estimated to account for 4% of visits to emergency departments,^1^ with a lifetime prevalence of about 25%.^2^, ^3^ Dizziness and balance diagnoses, however, are challenging from a clinical perspective given their multiple potential etiologies,^4^ and patients often seek care across multiple settings, including at urgent care centers, with primary care physicians, or with specialists in neurology or otolaryngology.^5^ Altogether, patients with dizziness or balance disorders can suffer a significant cost burden, with estimated annual medical expenditures for patients with dizziness and vertigo to exceed $48 billion.^6^

In addition to a clear impact on health care resource utilization, dizziness and balance disorders have a profound effect on the quality of lives of patients—patients suffering from dizziness report both significantly reduced mental and physical health‐related quality of life,^7^ even after adjusting for comorbitidites.^8^ These quality of life impacts may be even more pronounced among older patients,^9^ for whom dizziness can be particularly prohibitive in participating in activities of daily living.^10^ And even in younger adults, dizziness and balance disorders can negatively impact economic productivity—for example, many (70%) patients with vertigo report having to decrease their workload overall^11^ and even reported missing an average of over 2 weeks at work due to their diagnosis.^12^

In order to study the epidemiology of dizziness and balance disorders across adults in the United States, we used data from the National Health Interview Survey, which is conducted through personal household interviews by the United States Census Bureau in order to better understand health care access and health status on a national level. Our goal was to quantify the changes in prevalence and impact of dizziness and balance disorders in the adults in the United States from 2008 to 2016. While there are studies describing the epidemiology of dizziness at a single points in time,^13^ we sought to understand the changes over this time period to explore how health care resources could be better allocated in the future to improve both patient quality of life and decrease losses in economic productivity.

## Methods

The National Health Interview Survey (NHIS) data for the calendar years 2008 and 2016 were analyzed. Unique to these sample years, a specific balance survey module was contained within the NHIS. This module includes data on the prevalence of balance disorders, associated balance symptoms, and the impact of these disorders on self‐reported functional limitations. The study protocol was reviewed by the Mass General Brigham IRB and found to be exempt from review as it utilizes a publicly available, de‐identified data set.

The data set was restricted to sample adults, age ≥18.0 years. For each adult, demographic data and variable weights were extracted. First, the prevalence of reported dizziness or balance problems in the prior 12 months among adults was determined for each year and compared over time. Next, for each sample year, the prevalence of subtypes of dizziness/balance problems was determined with survey‐specific questions for difficulty with: unsteadiness, walking on uneven surfaces, faintness, and vertigo. Quantification of balance problem triggers and associated symptoms was obtained from the balance module for the potential trigger of prescription medications and for associated symptoms: nausea or vomiting, anxiety, and depression. Finally, the prevalence of functional limitations from balance problems was quantified, overall and for specific limitations in: exercise, driving, social interaction, and walking downstairs.

In order to understand time‐related differences in these aspects of balance problems, proportions were compared between the 2 sample years. As the NHIS utilizes a structured and weighted survey design, sample weights were applied to obtain nationally representative statistics. Results are reported as mean or proportion ± standard error of the national estimate, as appropriate. Statistical comparisons were conducted with *χ* ^2^ with statistical significance set at *p* = .05.

## Results

**Figure 1:**
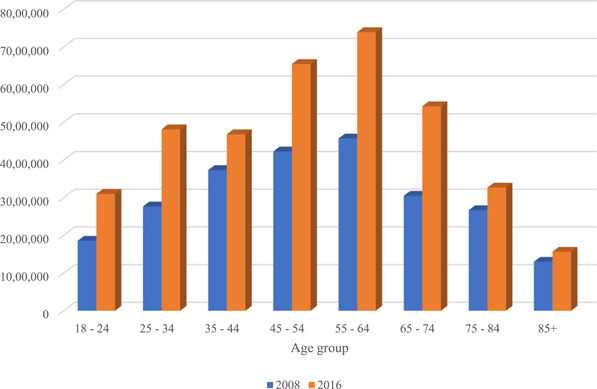
Number of adults reporting balance problem, 2008 versus 2016.

**Figure 2:**
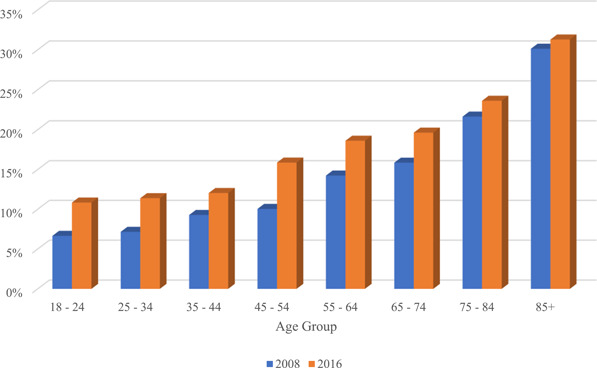
Percent of adults reporting balance problem, 2008 versus 2016.

A substantial number of adults in both survey years reported dizziness/balance issues: in 2016, 36.8 ± 1.0 million (15.5% ± 0.3%) adults (mean age, 52.4 years; 62.1% female) reported a balance problem in the prior 12 months, versus 24.2 ± 0.7 million (11% ± 0.3%) in 2008 (*p* < .001, Table 1). After adjustment for age and sex, this difference in prevalence remained significant (odds ratio 1.435 [1.332‐1.546], *p* < .001). Figure 1 depicts the increase in number of adults afflicted with dizziness/balance issues according to age group in 2008 and 2016, respectively, demonstrating an increase across all age groups; while this increase is likely in part attributable to population growth, Table 1 and Figure 2 demonstrate this is also due to a relative increase in the proportion of Americans reporting these tissues. Table 1 also demonstrates the symptom characterizations of these balance issues, including feeling off‐balance, faint, vertiginous, or having difficulty walking on uneven surfaces. Like the prevalence of dizziness overall, the proportion of patients who characterized their dizziness problem in these former 3 ways significant increased from 2008 to 2016 (*p* = .005, <.001, <.001, respectively).

**Table 1:** Epidemiology of Balance Disorder Symptoms in US Adults, 2008 Versus 2016

	2008				2016				
Symptom	N (millions)	SE (millions)	%	SE %	N (millions)	SE (millions)	%	SE %	Significance
Had a problem with dizziness or balance in the past 12 months	24.16	0.73	11.1	0.3	36.78	1.001	15.5	0.3	<0.001*
Felt off‐balance or unsteady	15.70	0.50	65.4	1.2	25.47	0.74	69.4	0.8	0.005*
Difficulty walking on uneven surfaces	9.17	0.38	38.0	1.1	14.84	0.52	40.4	0.9	0.101
Felt like fainting	9.67	0.39	40.3	1.2	17.78	0.59	48.5	0.9	<0.001*
Spinning or vertigo sensation with dizziness	9.42	0.37	39.3	1.1	16.81	0.58	45.9	0.9	<0.001*

Among these patients who reported balance issues, many reported associations of these symptoms with a prescription medication (20.7% and 19.2%) or nausea (24.6% and 27.1%) both in 2008 and 2016 (Table 2). Significantly more adults experienced anxiety (29.4% vs. 19.4%; *p* < .001) and depression (16.3% vs. 12.9%; *p* = .002) associated with their balance problems in 2016 versus 2008 (Table 2).

**Table 2:** Triggers and Associated Symptoms of Balance Disorders in US Adults, 2008 Versus 2016^a^

	2008				2016				
Associated trigger or symptom	N (millions)	SE (millions)	%	SE %	N (millions)	SE (millions)	%	SE%	Significance
Balance problem triggered by prescription medication	4.84	0.28	20.7	1.0	7.00	0.34	19.2	0.7	0.263
Nausea or vomiting with balance problem	5.87	0.27	24.6	0.9	9.9	0.44	27.1	0.9	0.056
Elevated anxiety with balance problem	4.54	0.25	19.1	1.0	10.70	0.45	29.4	0.8	<0.001*
Depression symptoms with balance problem	3.07	0.20	12.9	0.8	5.94	0.31	16.3	0.7	0.002*

^a^ Among those adults reporting a problem with balance in the prior 12 months.

Patients experienced substantial functional impact from their dizziness and balance issues both in 2008 and 2016 (Table 3). In the latter survey, adults with balance problems were limited in ability to drive motor vehicles (13.0%), exercise (14.4%), participate in social activities (12.9%), or walk downstairs (12.8%). However, these rates were not significantly different from 2008 (all *p* > .05).

**Table 3:** Reported Functional Impacts of Balance Problems in US Adults, 2008 Versus 2016^a^

	2008				2016				
Impact of balance problem	N (millions)	SE (millions)	%	SE%	N (millions)	SE (millions)	%	SE %	Significance
Balance problem prevents doing things	6.74	0.33	28.5	1.1	9.97	0.40	27.5	0.8	0.473
Balance problem affects ability to exercise	3.51	0.24	14.5	0.9	5.31	0.27	14.4	0.6	0.918
Balance problem affects ability to drive a motor vehicle	2.96	0.22	12.2	0.8	4.79	0.26	13.0	0.6	0.446
Balance problem affects ability to participate in social activities	2.7	0.20	11.3	0.8	4.7	0.24	12.9	0.6	0.114
Balance problem affects ability to walk downstairs	3.43	0.24	14.2	0.9	4.69	0.26	12.8	0.6	0.180

^a^ Among those adults reporting a problem with balance in the prior 12 months.

## Discussion

In this nationally representative analysis, we found a significantly increased prevalence of balance problems among adults and a significantly increased associated psychiatric symptom burden from 2008 to 2016. While the population is certainly aging, the increase in prevalence of balance problems was still significant even when adjusting for age and sex, suggesting other factors are likely involved. Obesity may play a role, as rates nationally rose from 33.7% to 39.6% in this time frame (2008‐2016).^14^ However, whether obese patients are more likely to suffer from balance problems is controversial—some studies have not found direct links between body mass index (BMI) and dizziness or vertigo, respectively,^15^, ^16^ while others suggest that increased BMI, especially for older women, may impair postural stability.^17^ Interestingly, among patients who experience dizziness, obese patients are more likely to experiences falls versus nonobese counterparts,^10^ as well as underestimate their own risk for falls.^15^ What may instead be playing a much more direct role in our results are effects from diabetes mellitus: peripheral neuropathy can certainly cause balance issues given impaired proprioception,^18^ and the national rate of diabetes (including types I and II) has increased significantly increased over the time period in our study (estimated from 13% to 34% from 2013‐2016 to 2020).^19^

What also may play a larger or more direct role in the increasing prevalence of dizziness is psychiatric pathology—our data clearly note a higher association between patients' balance issues and anxiety and depression. Given the phrasing of the question on the survey used, we cannot conclude if individuals' reported dizziness is underlying their psychiatric condition, or in fact anxiety or depression is instead driving a perceived balance issue. There exists strong evidence to suggest that psychosocial stress is linked in some way with dizziness^7^—in fact, vertigo is often seen comorbid with anxiety,^20^ and patients with panic disorder often report dizziness and can demonstrate vestibular dysfunction.^21^ However, the exact nature of the relationship between balance issues and psychiatric distress remains difficult to elucidate. While rates of anxiety in adults have increased in the United States over the last 10 years (5.1%‐6.7% from 2008 to 2018), although by only ~1.5%,^22^ rates of depression have not significantly changed (8.1%‐7.4% from 2007 to 2916).^23^ These data suggest that the dizziness/imbalance issues may be more leading to increased psychiatric symptoms given that the prevalence of dizziness/imbalance has increased at a faster rate than psychiatric comorbidities. We do note, however, that our study was limited to the diagnoses of anxiety and depression in assessing psychiatric pathology, and there certainly may be other disorders contributing to the morbidity of balance disorders for patients.

Regardless of etiology, the significant increase we found in the prevalence of dizziness/balance issues may require substantial health care resources in coming years, especially if this trend continues. Our data suggest an increasing prevalence advancing within an increasing overall population in the United States. The cumulative health care cost, already more than $48 billion,^6^ will only continue to grow, as patients seek care across the health care system, from emergency departments to specialists in the outpatient setting, often seeing multiple providers^5^ and often still lacking a definitive diagnosis and treatment plan.^4^ Dizziness is challenging symptom presentation, with etiologies ranging among cardiac, neurologic (including cerebrovascular), oto‐vestibular, respiratory, and metabolic or psychiatric origins.^4^ And while the etiologies of most presentation of dizziness may be appropriately treated as an outpatient, a subset of these patients will have a serious underlying etiology requiring urgent or emergent treatment,^24^ leading to significant resource use to distinguish these 2 groups: patients presenting to emergency departments with dizziness are more likely to have a longer emergency department stay, undergo more diagnostic testing and head imaging with computed tomography or magnetic resonance imaging, and be admitted to the hospital than nondizzy controls.^4^

Our finding that from 2008 to 2016 there was not a significant decrease in functional impact from dizziness symptoms suggests a lack of effect of our current management strategies to improve patients' quality of life over this time period. Alternatively, this finding may reflect the difficulty of patients to receive appropriate care, whether due to financial or other barriers. Even outside of the health care system, there is profound effect in terms of productivity of the US workforce with 1 in 7 adult‐age workers struggling with balance problems, leading to either missed work^12^ or decreasing workloads,^11^ which is only exacerbated given the difficulty many patients face in seeking and establishing care with a provider comfortable treating dizziness.

Our study does have limitations, as our data are drawn from 2 years of survey data and are self‐reported data, which may lead to an overestimation of symptom prevalence rates. Survey respondents may not necessarily carry a diagnosis of a formal balance disorder nor seek medical care for their balance symptoms they reported. The symptoms reported may be from a broad range of etiologies, and given they do not reflect specific diagnoses, this limits our ability to discern which areas of the US health care system may be most impacted. However, regardless of etiology, our data shows significant functional impact for patients due to these balance issues, highlighting the importance of these issues from a patient perspective. Future studies should focus on better understanding the relationship between psychiatric diagnoses like anxiety or depression and balance issues, as well as identify the drivers of health care costs for patients suffering from dizziness. Especially as the number of patients suffering from these issues grows, studies should also examine the efficacy of various treatment regimens in terms of lessening the functional impact of these symptoms to minimize their vast societal economic burden.

## Conclusion

In this nationally representative analysis, we found a significantly increasing prevalence of balance problems and a significantly increasing association of these balance problems with anxiety and depression from 2008 to 2016. Patients also noted substantial functional impacts, such as difficulty with their usual social interactions, exercise, driving a car, or walking downstairs due to their balance issues. In 2016, approximately 1 in 7 US adults experienced a dizziness or a balance problem. This issue certainly warrants more research, as likely changes in health care resource allocation should be considered in order to address anticipated growing health care costs, deficits in patient quality of life, and loss in economic productivity related to this symptom burden.

## Author Contributions

**Margaret B. Mitchell**, study design and conduct, analysis of data, drafting, revision, and final approval of manuscript; **Neil Bhattacharyya**, study design and conduct, analysis of data, drafting, revision, and final approval of manuscript.

## Disclosures

### Competing interests

None.

### Funding source

None.
